# Optical genome mapping identifies a homozygous deletion in the non-coding region of the SCN9A gene in individuals from the same family with congenital insensitivity to pain

**DOI:** 10.3389/fgene.2024.1375770

**Published:** 2024-08-02

**Authors:** Aïcha Boughalem, Viorica Ciorna-Monferrato, Natacha Sloboda, Amélie Guegan, François Page, Sophie Zimmer, Marion Benazra, Pascale Kleinfinger, Laurence Lohmann, Mylène Valduga, Aline Receveur, Fernando Martin, Detlef Trost

**Affiliations:** ^1^ Department of Human Genetics, Laboratoire CERBA SA, Saint Ouen L’aumône, France; ^2^ Génétique Médicale et Oncogénétique, Hôpital Femme Mère Enfant, CHR Metz-Thionville, site de Mercy, 1, Allée du Château, Metz Cedex, France

**Keywords:** optical genome mapping, *SCN9A* gene, insensitivity to pain, non-coding structural variant, structural variant detection

## Abstract

We report an index patient with complete insensitivity to pain and a history of painless fractures, joint hypermobility, and behavioral problems. The index patient descends from a family with notable cases among his maternal relatives, including his aunt and his mother’s first cousin, both of whom suffer from congenital insensitivity to pain. The patient had normal results for prior genetic testing: fragile-X syndrome testing, chromosomal microarray analysis, and exome sequencing. Optical genome mapping detected a homozygous deletion affecting the noncoding 5′ untranslated region (UTR) and the first non-coding exon of the *SCN9A* gene in all affected family members, compatible with recessive disease transmission. Pathogenic homozygous loss-of-function variants in the *SCN9A* gene are associated with impaired pain sensation in humans. Optical genome mapping can thus detect pathogenic structural variants in patients without molecular etiology by standard diagnostic procedures and is a more accessible diagnostic tool than short-read or long-read whole-genome sequencing.

## Introduction

To date, the panel of tools used for diagnostic genetic testing in patients suffering from rare or heterogeneous genetic diseases comprises conventional karyotyping, chromosomal microarrays (CMA) and recently next generation sequencing (NGS) for the sequence analysis of gene panels, and exome (ES) or genome (GS) analyses (Miller et al., 2010; Manickam et al., 2021).

Subsequent use of these techniques has greatly improved the diagnostic yield in genetic testing. However, each of the techniques used in human genetics has its own specific limitations, such as poor resolution for standard karyotyping, lack of structural information for CMA, and absence of sequence information for both techniques. Short-read sequencing approaches like ES or GS allow proper sequence analysis but have only limited pertinence for copy number variant (CNV) or structural variant (SV) detection (Álvarez-Mora et al., 2022).

Optical genome mapping (OGM) provides a new non-sequencing approach for a high-resolution analysis of the human genome. This technique involves the imaging of long linear single DNA molecules with a median size exceeding 250 kb. These molecules are labeled at specific sites and undergo *de novo* assembly, allowing the reconstruction of the patient’s genome. Subsequent structural variant calling is done by comparing the reconstructed genome to the reference genome (Lam et al., 2012).

We report three patients with complete insensitivity to pain (CIP). Despite extensive genetic analyses, including exome sequencing and chromosomal microarray (CMA) analysis, no definitive diagnosis was established in the index patient. However, optical genome mapping revealed a homozygous deletion within the non-coding region of the *SCN9A* gene, impacting the 5′ untranslated region (UTR) and encompassing the promoter, an enhancer element, and the first non-coding exon of the gene.

The *SCN9A* gene encodes the alpha subunit of the NaV1.7 sodium channel that is associated to human pain disorders (Cox et al., 2006; Baker and Nassar, 2020). Gain-of-function missense variants in *SCN9A* lead to extreme pain perception with autosomal dominant transmission (primary erythermalgia, OMIM 133020 or paroxysmal extreme pain disorder, OMIM 167400), whereas non-sense variants with subsequent loss-of-function NaV1.7 channels cause autosomal recessive congenital insensitivity to pain (insensitivity to pain, congenital CIP/neuropathy, hereditary, sensory, and autonomic type IID, OMIM 243000) (Hisama et al., 1993; Majeed et al., 2018).

Given these results, a combination of NGS sequencing and OGM techniques is promising for a further increase in the diagnostic yield in human genetic testing (Mantere et al., 2021; Neveling et al., 2021; Colin et al., 2023).

## Clinical description

We describe a Turkish family with multiple individuals affected by congenital insensitivity to pain (CIP).

The index patient (Figure 3: IV-5), a 7-year-old-boy, with an unremarkable perinatal history (birth weight: 3,370 g, birth length 50 cm, PC: 33, APGAR score: 9/10), was born at term. His 11-year-old brother and his 3-year-old sister are healthy.

At 15 months of age, insensitivity to pain was identified after an accident involving hot liquid, resulting in apparently painless burns on the face, neck, and body. By 18 months, psychomotor development was unremarkable, with normal comprehension (started walking at 8 months). However, the boy exhibited behavioral problems, such as head tapping and self-injury by biting, leading to multiple painless skull fractures.

At 3 years of age, impaired wound healing and joint hypermobility were observed. A neurological examination at 4 years of age indicated insensitivity to pain stimuli, normal nerve conduction (fibular nerves and sural nerve), normal pallesthesia, and normal electromyography (EMG).

Among the maternal relatives, the mother’s first cousin (Figure 3: III-10) presented with CIP along with behavioral issues, painless bone fractures, auto-mutilation, and impaired wound healing. The maternal aunt (Figure 3: III-6) had CIP and neuropathic joints. Two additional deceased individuals were reported to have CIP and hence were not available for genetic testing (Figure 3: I-1, II-5).

Among the paternal relatives, no cases of CIP have been reported. The paternal and maternal grandparents are first-degree cousins (Figure 3: II-1 and II-2, II-3, and II-4). No information concerning consanguinity is available for the great-grandparents, but both are from the same village.

The summary of clinical manifestations is given in Table 1.

**TABLE 1:** Clinical manifestations of the reported patients; (+) present, (−) absent, *nr* not reported, and CIP congenital insensitivity to pain.

Patient	CIP	Anosmia	Bone facture	Auto mutilation	Neuropathic	Poor wound healing
					joint	
Index	+	+	+	+	+	+
Great uncle	+	+	+	+	+	+
Aunt	+	+	−	−	+	−
Deceased 1	+	nr	nr	nr	nr	nr
Deceased 2	+	nr	nr	nr	nr	nr

Given the clinical presentation and behavioral issues, genetic counseling was provided, which recommended psychiatric follow-up and care.

The affected patients have undergone genetic counseling with the referring clinical geneticist; all individuals analyzed gave their consent for genetic testing and publication, and for minors participating as probands, written informed consent was obtained from their parents.

## Materials and methods

For each sample, 650 µL of whole peripheral blood or 1.5 million cultured cells were used to purify ultra-high molecular weight DNA using the SP Blood and Cell Culture DNA Isolation Kit following the manufacturer’s instruction (Bionano Genomics, San Diego, United States). Briefly, after cell counting, white blood cells were pelleted (2200 g for 2 mn) and treated with LBB lysis buffer and proteinase K to release genomic DNA (gDNA). After the inactivation of proteinase K by PMSF treatment, genomic DNA was bound to a paramagnetic disk, washed, and eluted in an appropriate buffer. Ultra-high molecular weight DNA was left to homogenize at room temperature overnight.

DNA molecules were labeled using the DLS (Direct Label and Stain) DNA Labeling Kit (Bionano Genomics, San Diego, United States). Seven hundred and 50 nanograms of gDNA was labeled in the presence of direct label enzyme (DLE-1) and DL-green fluorophores. After clean-up of the excess of DL-Green fluorophores and rapid digestion of the remaining DLE-1 enzyme by proteinase K, the DNA backbone was counterstained overnight before quantitation and visualized on a Saphyr instrument.

A volume of 19.5 µL of labeled UHMW gDNA solution with a concentration between 4 and 12 ng/μL was loaded on a Saphyr chip and scanned using the Saphyr instrument (Bionano Genomics, San Diego, United States). The Saphyr chip ran at a target of 400 Gbp to aim for 100X coverage. All samples met the recommended metrics (effective coverage, map rate, N50, and label density).

The *de novo* assembly and variant annotation pipeline were executed on Bionano Solve software V3.7. Reporting and direct visualization of structural variants were done on Bionano Access V1.7.2. Recommended filtering was used and corresponds to the following minimum confidence values: insertion/deletion = 0, inversion = 0.7, duplications = −1, intra-translocation = 0.3, inter-translocation = 0.65, and CNV = 0.99. Additional filtering by pairwise comparison against the parents’ assemblies (trio analysis) was performed to easily distinguish inherited and *de novo* variants in the proband.

## Results

Genetic testing of the index patient using chromosomal micro-array (CMA), fragile-X FMR1 testing, and exome sequencing provided non-conclusive results.

Optical genome mapping detected 25 insertions, 33 deletions, and 1 inversion; these SVs were not identified by prior NGS analysis. Of the detected SVs, 33 were overlapping with an OMIM disease gene. The respective genes were correlated to the patient’s phenotype to identify candidate disease genes.

**FIGURE 1:**
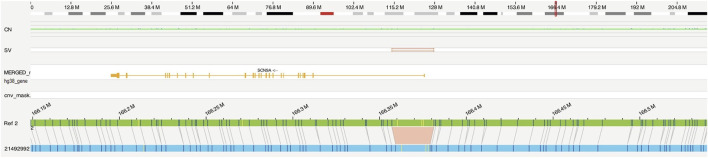
Optical genome mapping identified a homozygous 4.3-kb deletion, probably affecting the 5′ UTR and the first exon of the *SCN9A* gene (ogm[GRCh38]2q24.3(166357064_166381268)x0). Optical map of the patient in blue, and the reference of chromosome 2 in green.

**FIGURE 2:**
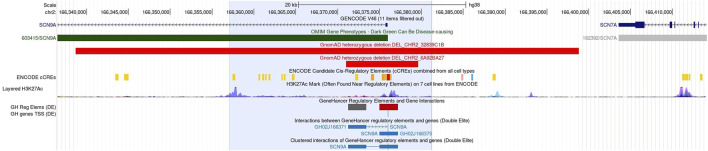
UCSC detail of the genomic region (24 kb, blue shade) containing the homozygous 4.3-kb deletion, and the exact position of the deleted segment is unknown. In this non-coding region, control elements of the *SCN9A* gene are located: first exon, promoter, and enhancer elements (Nassar et al., 2023). No homozygous benign CNVs are known in this area, and heterozygous losses from GnomAD are given in light red bars.

OGM identified a homozygous 4.3-kb deletion, flanked by two OGM DNA labeling sites at 24 kb distance, affecting the 5’ UTR of the *SCN9A* gene (Figure 1), including the promoter and an enhancer element, as well as the first non-coding exon of the gene: ogm[GRCh38] 2q24.3(166357064_166381268)x0 (Figure 2). Notably, this deletion is absent in the 179 OGM control samples. Moreover, the Database of Genomic Variants (DGV) does not report any benign copy number variations in this region (MacDonald et al., 2014).

## Segregation analysis

**FIGURE 3:**
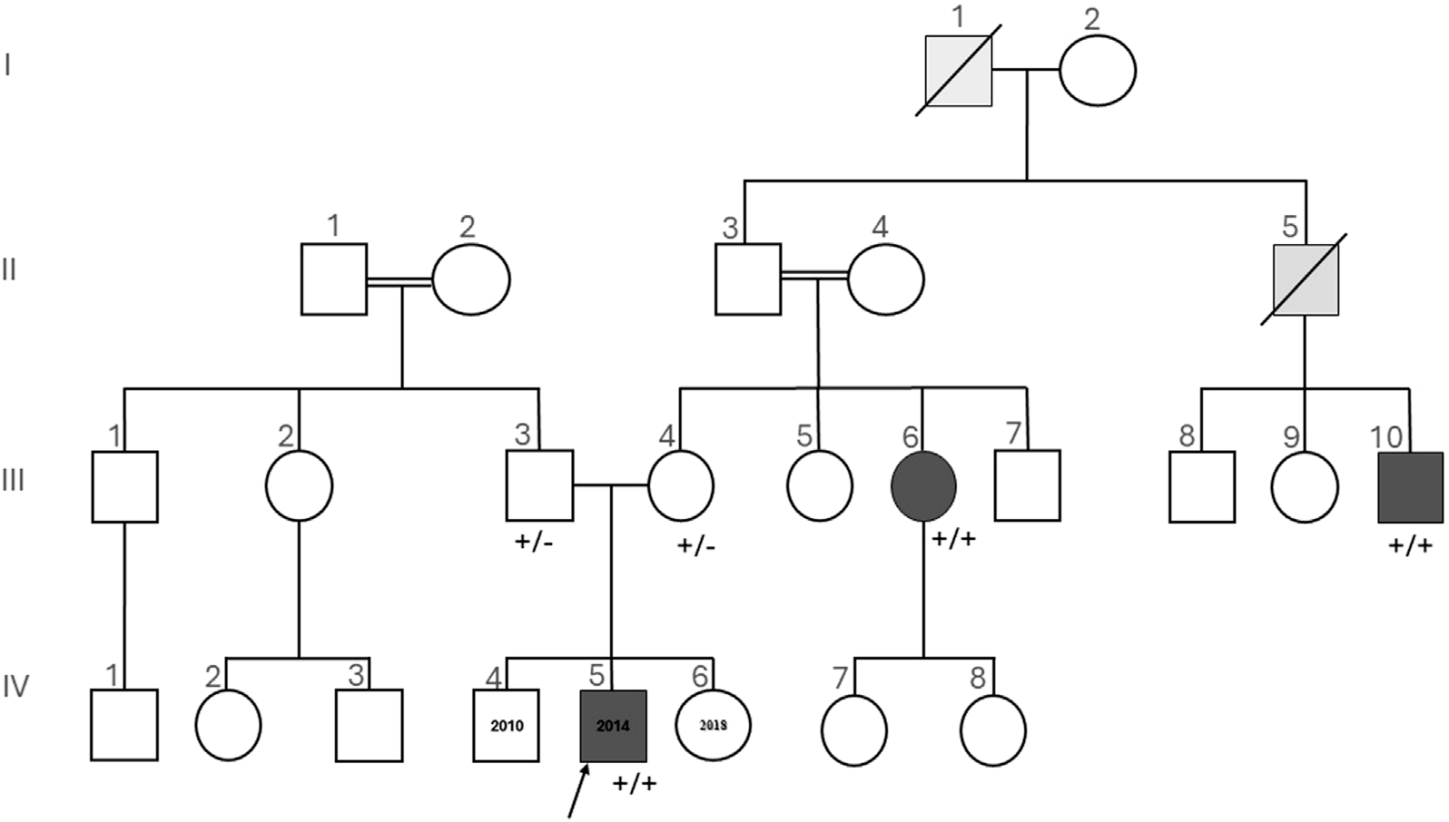
Pedigree of the investigated family, which is of Turkish descent. Squares are used for male members, circles for female members, and slashes for the deceased. The proband (IV-5) is indicated by the arrow. Dark gray symbols show members with both insensitivity to pain and anosmia. Light gray symbols show members with insensitivity to pain, not tested for anosmia. +/+ homozygous or +/− heterozygous indicates the status of *SCN9A* given for the tested individuals. Insensitivity to pain and anosmia co-segregates with homozygosity for the described *SCN9A* deletion.

Both parents were found to be heterozygous carriers of the same deletion (Figure 3: III-3, III-4). Homozygosity of this deletion co-segregates with CIP in two additional affected individuals tested from the same family, namely, the aunt (Figure 3: III-6) and the mother’s first cousin (Figure 3: III-10).

## Reverse phenotyping

Reverse phenotyping in the affected individuals revealed complete anosmia in all cases.

## Variant classification

Following the American College of Medical Genetics and Genomics (ACMG) classification for CNVs (Riggs et al., 2020), the patient variant in the *SCN9A* gene has been classified as probably pathogenic (0.9) based on the following criteria: 1A, 2B, 2C, 3A, 4F, and 5D.

## Discussion

In this study, we describe a family with three individuals presenting with complete insensitivity to pain (CIP), painless fractures, and anosmia as the key clinical symptoms. All three affected family members carry a novel homozygous deletion probably (Karczewski et al., 2020) affecting the non-coding 5′ UTR and the first exon of the *SCN9A* gene. Heterozygous carriers in this family having the same deletion are unaffected. The families of both parents of our index patient are of Turkish origin, and both families live in the same area of the country. The parents of our index patient have no information on the possible consanguinity of their families, and both parents emigrated independently from Turkey. The 5’ variant detected using OGM has not been observed in the OGM control population (n = 179) [Bionano Solve Theory of Operation: Variant Annotation Pipeline] and has not been observed in analyses of other patients in our laboratory (n = 150). No homozygous CNVs are reported in the reference databases GnomAD, ClinVar, and DGV [12, 14, and 18]. Heterozygous CNVs are illustrated in Figure 2. Despite no known consanguinity between the two branches of the investigated family, the appearance of a rare variant in multiple family members is suggestive of a population with consanguineous origin.

This deletion may lead to the loss of function in the *SCN9A* gene. The loss-of-function variants in the *SCN9A* gene are associated with CIP, whereas the gain-of-function variants cause extreme pain perception (Baker and Nassar, 2020).

The reported gain-of-function mutations in patients cause a lower activation threshold in primary erythermalgia (PEM, OMIM 133020); thus, the Nav1.7 channel is more easily activated. In paroxysmal extreme pain disorder (PEPD, OMIM 167400), the gain-of-function variants result in a higher inactivation voltage of Nav1.7, and the channel inactivation is incomplete (Baker and Nassar, 2020).

It has been shown that *SCN9A* and *NTRK1* are the major genes for CIP. The secondary symptoms associated with the respective genes differ between *SCN9A* (anosmia) and *NTRK1* (anhidrosis) (Lischka et al., 2023). No interaction is known between these two genes or the other minor genes implicated in CIP.

An indirect interaction between *SCN9A* and *CACNA2D1* may exist through LAT1 (*SLC7A5*) as a common regulator of both; indeed, CIP can be present in patients with *CACNA2D1* variants as a part of a more syndromic and severe neurodevelopmental disorder (Dahimene et al., 2022). The exact interactions between these genes are to date unknown (Alles et al., 2020).

Homozygous loss-of-function variants in the *SCN9A* gene were initially associated with the inability to experience pain in three consanguineous families from northern Pakistan.

Beyond pain insensitivity, one of the patients displayed behavioral particularities as he placed knives through his arms and died jumping off a house roof. Self-injuries were reported in all affected patients (Cox et al., 2006). Recently, new patients with homozygous variants in *SCN9A* affected with CIP have also been reported to have behavioral phenotypes (Romagnuolo et al., 2023).

In the present study, both the index patient (Figure 3: IV-5) and his great uncle (Figure 3: III-10) display behavioral problems including restlessness and head tapping, which caused several painless skull fractures during childhood.

As another key symptom, individuals with *SCN9A*-associated CIP were found to be completely anosmic (Weiss et al., 2011). Although this feature had not been reported initially in the investigated patients, a clinical reassessment with targeted testing, however, confirmed that the patients indeed were completely anosmic, thus adding pertinent clinical information. To our knowledge, no other homozygous non-coding variants have been reported in the literature or in the HGMD database for any genes associated with anosmia (Stenson et al., 2003; Kamarck et al., 2024).

Targeted clinical re-evaluation after genetic testing of previously unreported patient phenotypes greatly improves the diagnostic yield (Barili et al., 2023).

The presented deletion probably damages the 5’ UTR and the first exon of *SCN9A*. In this region, several control elements of the gene have been identified (Figure 2). The exact position of the 4.3 kb deletion within the region of 24 kb delimited by the flanking OGM marker is, however, unknown, and its effect on the gene regulation is not clear. This region neither contains any reported pathogenic sequence variants nor pathogenic CNVs in DECIPHER or ClinVar (Firth et al., 2009; Landrum et al., 2018). The region is, however, non-polymorphic in the DGV database (MacDonald et al., 2014). Functional studies for this deletion are hampered by the lack of more precise positional information. mRNA testing with a skin biopsy might provide further evidence for the effect of the 5’ deletion on *SCN9A* gene expression. Following the ACMG classification for CNVs, the presented deletion is probably considered pathogenic (Riggs et al., 2020).

The reported deletion in our family affects a non-coding region, and the contributions of most non-coding copy numbers and structural variants to human diseases are unclear, yet most of the genome-wide association studies (GWAS) signal maps to non-coding regions (Zhang and Lupski, 2015). In addition, the classification of new unknown variants detected using novel diagnostic tools is often challenging. The most powerful evidence for pathogenicity is phenotype correlation and segregation analysis.

A homozygous deletion in the non-coding part of the *SCN9A* gene, probably affecting control elements of this gene, co-segregates with the expected disease phenotype, as found in this study. This finding is a strong argument for the pathogenicity of this variant. To the best of our knowledge, this is the first description of a non-coding, probably pathogenic variant affecting *SCN9A*.

The novel diagnostic tool OGM offers distinct advantages compared to CMAs, as it provides a comprehensive view of the genome; can identify complex structural variations that may be missed by traditional genotyping methods, including balanced structural variations that remain undetected with CMA; and additionally allows the determination of the underlying chromosomal mechanism [3, 23, and 24]. Although short-read whole-genome sequencing has provided valuable insights, particularly in identifying exonic deletions affecting *SCN9A* (French et al., 2022), it is important to note its limitations, especially regarding the detection of structural variants (de Bruijn et al., 2022). In addition to these challenges, routine diagnostic tools, such as exome sequencing (Twist Bioscience, South San Francisco, California, United States) and chromosomal microarray (CytoScan HD Array by ThermoFisher Scientific, Waltham, Massachusetts, United States), used in our case, failed to cover the 5′ UTR region and the first exon of the *SCN9A* gene due to the absence of probes in this region.

This highlights the pressing need for new diagnostic approaches to enhance the effectiveness of genetic testing and uncover comprehensive genomic information (Wojcik et al., 2023).

The presented 5′ deletion is a candidate variant that is responsible for painlessness in the investigated family. The sharing of new candidate variants has been of great utility for the analysis of genomic data. For sequence variants, classification guidelines (ACMG/AMP) are used for the reporting of new variants detected using exome or genome sequencing, and this greatly improved the reporting of genetic variants for rare diseases. For genomic structural variants until now, no classification guidelines are available; however, we feel that there is sufficient evidence to take the 5′ *SCN9A* deletion into consideration as the phenotype of our patients, and the reported *SCN9A* cases are nearly perfectly matching (clinical gene–disease validity). *SCN9A* is a known OMIM-listed disease gene associated to pain insensitivity (Gene knowledge). The 5’ deletion is probably located in an interval harboring elements indispensable for gene regulation and gene function (promoter, enhancer, and first exon) [Figure 2].

The sharing of this 5’ *SCN9A* variant might facilitate the detection of other non-coding variants affecting *SCN9A* in patients with impaired pain perception (Chong et al., 2024).

The ability of optical genome mapping to accurately reconstruct the patient’s genome is the key argument for performing OGM for patients with unresolved genetic diseases. This holds true even after undergoing diagnostic exome or genome sequencing and chromosomal microarray analysis (Chen et al., 2020; Mantere et al., 2021; Wojcik et al., 2023).

## Data Availability

The datasets for this article are not publicly available due to concerns regarding participant/patient anonymity. Requests to access the datasets should be directed to the corresponding author.
